# Celiac Disease and Thrombotic Events: Systematic Review of Published Cases

**DOI:** 10.3390/nu14102162

**Published:** 2022-05-23

**Authors:** Nikola Pantic, Ivana Pantic, Dorde Jevtic, Vanajakshi Mogulla, Stevan Oluic, Momcilo Durdevic, Terri Nordin, Mladen Jecmenica, Tamara Milovanovic, Tatjana Gavrancic, Igor Dumic

**Affiliations:** 1 Clinic of Hematology, University Clinical Center of Serbia, 11000 Belgrade, Serbia; drnikolapantic@gmail.com; 2 Clinic of Gastroenterology and Hepatology, University Clinical Center of Serbia, 11000 Belgrade, Serbia; ilic.ivana04@gmail.com (I.P.); tamara.alempijevic@med.bg.ac.rs (T.M.); 3 Elmhurst Hospital Center, Department of Internal Medicine, Elmhurst, NY 11373, USA; djordje965@gmail.com; 4 Icahn School of Medicine at Mount Sinai, New York, NY 10029, USA; 5 Mayo Clinic Alix School of Medicine, Rochester, MN 55905, USA; vanajakshi.mogulla@mayo.edu (V.M.); nordin.terri@mayo.edu (T.N.); 6 Department of Hospital Medicine, Mayo Clinic Health System, Eau Claire, WI 54703, USA; 7 Department of Internal Medicine, Loyola University Medical Center, Maywood, IL 60153, USA; stevan.oluic@luhs.org; 8 Department of Hospital Medicine, Advocate Aurora Health, Green Bay, WI 54311, USA; momcilo379@gmail.com; 9 Oceana Gastroenterology Associated, Corona, CA 92881, USA; giomla@gmail.com; 10 Department of Hospital Medicine, Mayo Clinic, Jacksonville, FL 32224, USA; gavrancic.tatjana@mayo.edu

**Keywords:** gluten-sensitive enteropathy, celiac disease, thrombotic events, thromboembolism, thrombosis, hypercoagulability, extraintestinal celiac manifestations

## Abstract

Extraintestinal manifestations of celiac disease (CD) should be considered, even in patients without typical intestinal symptoms. The aim of our study is to examine the literature regarding the occurrence of thrombotic events in CD, and to synthesize the data from case reports and case series. A systematic review of the literature was conducted by searching the Pub-Med/MEDLINE database, from the date of database inception to January 2022, to identify published cases and case series on this topic, in accordance with the PRISMA (Preferred Reporting Items for Systematic Reviews and Meta-Analyses) guidelines. A total of 55 cases were included in the study. The majority of patients were previously healthy individuals, with no comorbidities. In less than one-third of the cases (30.91%), the diagnosis of CD was established before the onset of thrombosis, while in the remaining cases (34.54%), thrombosis preceded the diagnosis or was diagnosed concomitantly with CD. The most common sites for thrombosis occurrence were hepatic veins (30.91%), while thrombosis of cerebral blood vessels, deep venous thrombosis of lower extremities, and pulmonary thromboembolism were less frequent. Thrombosis was most commonly isolated to one site only (78.18%). In 69.09% of cases (*n* = 38), some form of anticoagulation, along with a gluten-free diet, was initiated.

## 1. Introduction

There are various extraintestinal manifestations of celiac disease (CD), which are common and sometimes more prominent than readily recognized intestinal symptoms and signs, such as abdominal pain, chronic diarrhea, and malabsorption [1,2,3]. Cutaneous (dermatitis herpetiformis), hepatic (celiac hepatitis), neurologic (gluten ataxia), and musculoskeletal (arthritis and myopathies) manifestations are well recognized and widely described [1,2,3,4]. It has also been recognized that CD is associated with other autoimmune diseases, such as type 1 diabetes mellitus and autoimmune thyroiditis [1,2,3]. Hematologic abnormalities in patients with CD include anemia, thrombocytosis, thrombocytopenia, lymphoma, IgA deficiency, hyposplenism, and hypercoagulability [5,6,7]. Hypercoagulability has only recently emerged as a manifestation and/or complication of CD. The evidence of hypercoagulability in CD was first reported in the forms of case reports and case series [8,9]. Since then, multiple other reports appeared to suggest the connection [10,11,12], although various observational studies and meta-analyses have found conflicting results [13,14,15,16,17]. A number of well-designed prospective studies are ongoing in an attempt to understand this association and provide definitive answers [18].

The goal of our study is to examine the literature on this topic and synthesize the data from case reports and case series.

## 2. Materials and Methods

A systematic review of the literature was conducted by searching the PubMed/MEDLINE database, from the date of database inception to January 2022, to identify published cases and case series of patients with CD and thrombotic events (TE), in accordance with the PRISMA (Preferred Reporting Items for Systematic Reviews and Meta-Analyses) guidelines. A combination of the following key words was used: “celiac disease”, “coeliac disease”, “gluten-sensitive enteropathy”, “gluten hypersensitivity”, “non-tropical sprue”, “thrombosis”, “thromboembolism”, “pulmonary embolism”, and “hypercoagulability”.

**Figure 1:**
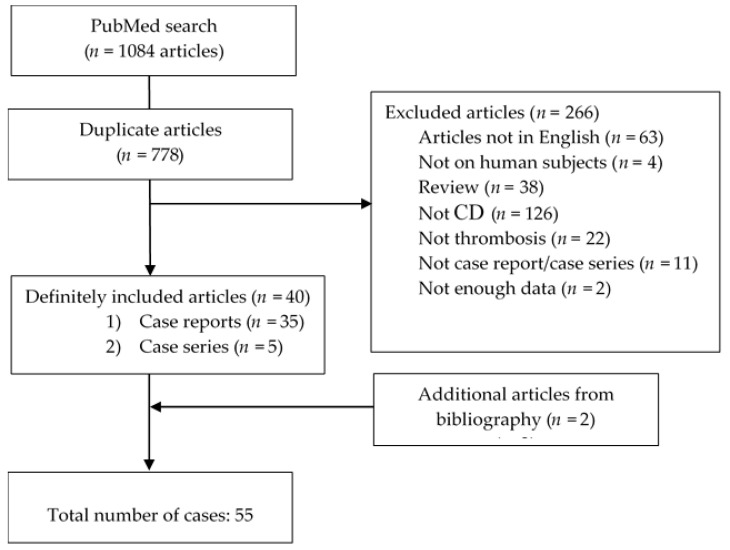
PRISMA flow chart.

The search was independently performed by two authors (N.P. and I.P.). Discrepancies that occurred during the selection process were resolved by consensus with the senior author (I.D.). We included case reports and case series with biopsy-proven CD and documented TE. Duplicate articles, narrative review articles, articles written in a language other than English, and cases that lacked small-bowel biopsy were also excluded. After the search was conducted, a total of 1084 articles were identified, out of which 778 were duplicates. References of the selected articles were further manually searched, and 2 additional articles were identified. The selection process resulted in the inclusion of a total of 42 articles in this study. The final number of cases included in this review is 55. PRISMA flow chart is illustrated below (Figure 1).

Afterwards, an Excel sheet was created using the following data for each case: PubMed Identifier (PMID), name of the first author, publication year, patients’ demographic data, co-morbidities, site of thrombosis, complete blood count parameters, presence of any hemostatic abnormalities and associated gastrointestinal manifestations, temporal relation between CD and thrombosis occurrence, thrombotic risk factors, presence of thrombophilic disorders, treatment modalities and final outcome.

## 3. Results

### 3.1. Demographic Characteristics and Comorbidities

In the cases published to date, the median age of patients was 32 years (range 8–66), predominantly females (*n* = 34, 61.82%), and the majority were previously healthy, with no comorbidities (*n* = 36, 65.45%), as can be observed in Table 1. When present, the reported comorbidities (which might further increase the risk of TE development) included single cases of each of the following: cardiomyopathy, cirrhosis, chronic obstructive lung disease, myeloproliferative neoplasm, autoimmune hepatitis, inflammatory bowel disease, membranous glomerulonephritis, and rheumatoid arthritis.

### 3.2. Clinical Presentation and Laboratory Parameters

Almost one-third of these patients with CD developed thrombosis of hepatic veins (*n* = 17, 30.91%), therefore making it the most common thrombosis site. However, thrombosis of cerebral blood vessels, deep venous thrombosis (DVT) of lower extremities, and pulmonary thromboembolism (PTE) were not uncommon (*n* = 10, 18.18%; *n* = 10, 18.18%; and *n* = 9, 16.36%, respectively). Less common thrombosis sites (defined as “other” in Table 2) included the following: coronary arteries, subclavian vein, dermal and placental blood vessels, and superficial blood vessels of lower extremities. TEs were mainly venous (*n* = 44, 80%), but in two cases, concurrent arterial and venous thrombosis were described [19,20]. Thrombosis was most commonly limited to only one site (*n* = 43, 78.18%). When it comes to presenting symptoms, abdominal symptoms were most commonly reported (*n* = 26, 47.27%), often in combination with symptoms related to the thrombosis. In less than one-third of cases (*n* = 17, 30.91%), the diagnosis of CD was established before the onset of thrombosis. Interestingly, in the remaining cases, thrombosis preceded the diagnosis of CD (34.54%) or was diagnosed concomitantly with CD during the diagnostic work-up for etiology of thrombosis (34.54%). The time between CD diagnosis and thrombosis occurrence (in cases when CD was diagnosed first) ranged greatly, from several days to several decades. When it comes to the initial laboratory parameters, anemia was recorded in half of the cases (*n* = 29, 52.72%). Thrombocytosis and thrombocytopenia were noted in 6 (10.91%) and 4 (7.27%) respectively.

### 3.3. Risk Factors for Development of Thrombotic Events

**Figure 2:**
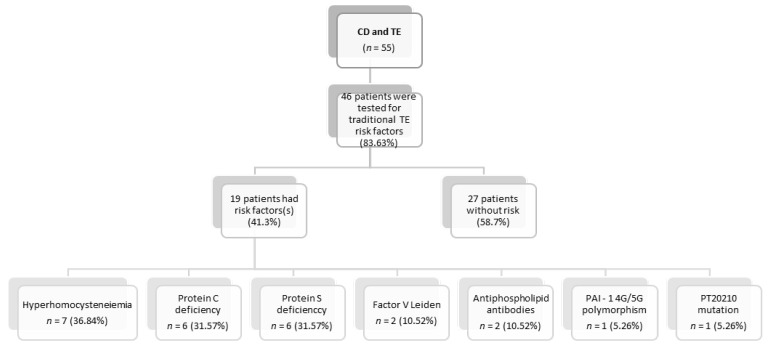
Thrombophilia in patients with celiac disease and thrombotic events.

Screening for thrombophilia was performed in 83.63% of the reported cases (*n* = 46), and concomitant thrombophilia was detected in more than one-third of the cases (*n* = 19, 41.4%), with hyperhomocysteinemia being the most common form (*n* = 7, 36.84%), followed by protein C and protein S deficiency (*n* = 6, 31.57%, each). In the remaining 58.7% of cases, no other thrombotic risk factors, apart from CD, were discovered (Figure 2).

### 3.4. Treatment Modalities and Outcome

In 69.09% of cases (*n* = 38), some form of anticoagulation was initiated. Vitamin K antagonists were the most common treatment of choice, and were administered in half of the cases (*n* = 19), either alone or as an extension of low-molecular-weight heparin treatment. The duration of both anticoagulation and antiplatelet treatment was not specified in the majority of cases. Out of those reported, it ranged from three months to lifelong. More than half of the patients totally recovered (*n* = 29, 52.72%). A lethal outcome was not uncommon, and occurred in 14.54% of cases (*n* = 8). The results are presented in detail in Table 1 and Table 2.

## 4. Discussion

### 4.1. Epidemiology and Demographic Characteristics

CD is a multifactorial disease. It develops in genetically susceptible individuals carrying the HLA-DQ2 or HLA-DQ8 haplotype, who, in response to unknown environmental factors, develop an immune response to dietary gluten [21,22]. However, this genetic predisposition to the development of CD is only partially explained by currently identified genes. Therefore, it has been suggested that epigenetics could have an equally important role in CD pathogenesis [21]. The estimated prevalence of CD in the general population is around 1% [23]. Most of these data come from western countries, while the observed lower prevalence of 0.4–0.6% in Asia, Africa, and South America might be due to under-reporting and a lack of population-based studies in these regions [24,25]. The Saharawi people of Algeria, who represent 22% of our cases, have some of the highest rates of reported CD seroprevalence (5.6%), which is thought to be due to a high frequency of the HLA DR3-DQ2 haplotype, high degree of consanguinity, reduced breastfeeding rate, and early gluten consumption in childhood [26,27,28]. The incidence of CD has been increasing by 7.5% per year over the last several decades, across many groups, regardless of age or sex [24]. The rising incidence can partially be explained by the introduction of more sensitive serologic tests, implementation of screening strategies in high-risk patients, and an overall increase in awareness of the disease and its atypical manifestations [24]. Environmental factors, such as socioeconomic differences, increased gluten consumption after infancy, and decreased rate of infections, which leads to an increased rate of autoimmune diseases (i.e., the hygiene hypothesis), contribute to this observed increase in the incidence of CD [29]. Our review found that thrombotic complications were significantly more common in female patients, with almost twice as many reported in the literature. These complications occurred at a younger age than in their male counterparts. Differences in incidence among sexes can be explained by increased health awareness and healthcare utilization, as well as increased prevalence of other autoimmune diseases that are more common in females and often coincide with CD [24]. Another possible explanation is the higher prevalence of thrombosis risk factors, such as the use of oral contraceptives and pregnancy, which were present in 13% of patients in our review. In the pediatric population, the higher incidence is explained by the greater tendency for CD to be symptomatic and present as classic CD in children, while adult patients can often present with a more indolent course, and as less typical or subclinical CD [30].

### 4.2. Clinical Presentation

Individuals who are sensitive to gluten have various intestinal and extraintestinal manifestations, which are commonly defined under the umbrella term “gluten-related disorders” [31]. When it comes to typical clinical manifestations of CD, those most commonly reported are abdominal symptoms (abdominal pain, diarrhea, bloating etc.) and signs and symptoms of malabsorption (steatorrhea, weight loss, nutrient and vitamin deficiencies). In this study, abdominal symptoms were reported in almost half of the cases (47.27%), while weight loss and growth restriction were probably underreported, as they were noted in only 10.9%. Since this review included patients with CD who developed a thrombotic event, it is not surprising that the vast majority of the other clinical symptoms reported (such as neurological symptoms and swelling of lower extremities, described in 21.81% and 14.54%, respectively) were actually a consequence of the thrombotic event, rather than CD itself.

Hematologic abnormalities in CD are common and include anemia, observed in up to 69% of patients (microcytic due to iron deficiency (IDA), or macrocytic due to B12 or B9 deficiency), and thrombocytosis in up to 60% of patients [6]. The reported data are in line with the results from this review, since the most observed hematologic abnormality was anemia, noted in more than half of the patients with CD (52.72%) [6]. Other hematologic abnormalities associated with CD are coagulopathy, thromboembolism and thrombocytopenia. When present, thrombocytopenia is usually associated with other autoimmune manifestations that are common in patients with CD [6].

### 4.3. Thrombosis and Celiac Disease

There is conflicting evidence regarding the risk of thrombosis in CD, which is uncommon and has been seldom reported in the literature. One cross-sectional study did not demonstrate increased risk; however, the results were limited by a relatively small study sample [32]. On the other hand, a recent meta-analysis reported a 25% increased risk of developing venous thromboembolism (VTE) in patients with CD, compared to patients without CD [17]. Similarly, the study by Ludvigsson et al. found an increased risk of VTE in the adult population with CD, which did not differ significantly between sexes [16]. While VTE can present before or after the diagnosis of CD, we found that VTE more commonly preceded CD manifestations or was diagnosed at the same time as the CD (in 34.54% patients each). In patients without traditional VTE risk factors, who develop VTE (especially in younger patients), CD might be the sole underlying cause of VTE, while in those who already have one of the well-recognized risk factors for VTE, CD might further contribute to VTE development.

The diagnosis of CD can, however, be missed on the initial assessment, as was evident in one female patient who presented with cerebral vein thrombosis more than a year after initial thrombotic event, only to be appropriately diagnosed after cerebral vein thrombosis occurred for the second time. Sometimes, the delay in diagnosis can be so severe that patients experience multiple complications, including severe IDA, heart failure, dilated cardiomyopathy, and DVT, as was evidenced in an 18-year-old in whom CD diagnosis was delayed for more than 5 years [33].

Thrombosis in CD can be arterial, venous, or concurrent, and can affect one or multiple blood vessels. Arterial thrombosis in CD is uncommon and most of the studies have previously assessed the risk of VTE [16,17,32]. Our review finds that venous thrombosis is significantly more common, affecting 80% of the patients, with hepatic vein thrombosis (Budd–Chiari syndrome) being the most common form (30.91%). Sometimes, in cases of Budd–Chiari syndrome, thrombosis extends to inferior vena cava, which increases the risk of PTE. This risk seems to be especially high if patients do not adhere to a gluten-free diet (GFD) [9,34,35]. DVT of lower extremities is also common (18.18%), typically presenting as swelling in 80% of patients and sometimes complicating with PTE, which was present in 16.36% of our cases [36,37].

### 4.4. Hypothesis Regarding Pathogenesis of Thrombosis in CD

Several possible factors could be associated with the higher risk of thromboembolic events in patients with CD.

Firstly, malabsorption caused by CD often leads to vitamin deficiencies (vitamin K, B6, B12, and folate deficiencies) [17,38]. Vitamin K, as a coenzyme in glutamic acid carboxylation, is a cofactor for the synthesis of protein C and its cofactor protein S [39]. Deficiency of protein C and protein S leads to uncontrolled coagulation activation and consequential thrombosis [40]. We find that, in this cohort, protein C and S deficiency was present in 31.6% of patients. On the other hand, vitamins B12, B6 and folic acid are essential for homocysteine metabolism [17,41,42]. Homocysteine is synthetized from methionine and could be converted back to methionine or transformed into cysteine. Deficiency of vitamins B12, B6 and folic acid interrupts this metabolic pathway, which leads to hyperhomocysteinemia [42]. Hyperhomocysteinemia could be present in individuals with a C677T mutation in the MTHFR gene. However, this mutation does not occur more in individuals with CD than in the healthy population [41]. Hyperhomocysteinemia is connected to different procoagulant states, such as platelet activation, endothelial dysfunction, oxidative stress, and reduced levels of protein C and antithrombin [43]. Hyperhomocysteinemia was also present in a substantial number of cases included in our review (36.8%).

Venous thromboembolism is frequently associated with autoimmune diseases, although the exact mechanism is still unknown [44]. The study published by Zöller et al. showed that individuals with autoimmune disorders had a significantly increased risk of PE within the first year after diagnosis [14]. This could be explained by chronic inflammation, which is a main feature of autoimmune diseases [44,45]. Interleukin-6 increases the expression of the tissue factor on monocytes, while interleukin-1 and TNF-α interfere with the activity and transcription of thrombomodulin. Moreover, plasminogen activator inhibitor-1 is increased in the state of chronic inflammation, therefore resulting in lower conversion of plasminogen to plasmin, and the consequential inhibition of fibrin degradation. Protein C activity has also been reported to be reduced in proinflammatory conditions, while fibrinogen (as an acute-phase reactant) is increased [44]. Chronic inflammation contributes to the development of atherosclerotic plaques and consequential endothelial dysfunction and thrombosis [46].

One of the most studied examples among autoimmune gastrointestinal diseases and thrombosis is inflammatory bowel disease (IBD). In a case–control study, the prevalence of IBD was found to be tenfold in CD cases; meanwhile, the prevalence of CD in IBD cases was comparable with that of the controls [47,48]. The increased prevalence of IBD in CD may be due to the common immune pathogenesis of both diseases resulting in inflammation, and by sharing genetic and environmental factors [49]. It may also be likely that the inflammatory response caused by CD may trigger pathophysiological events that lead to the development of IBD. This could be due to defects in the mucosal barrier [50], caused by CD increasing the intestinal permeability, which eventually allows unprocessed antigens to interact with the immune system, leading to an abnormal immune response and inflammation in IBD [51,52]. In this patient series, IBD was reported in two patients (3.64%). It has been reported that patients suffering from IBD carry at least a three times higher risk of venous thromboembolism when compared with the general population [53]. It has also been shown that there is a statistically significant difference between IBD patients and controls in thrombocyte volume, protein C, protein S, APC resistance, F1+F2 fragments, and tPA, which are associated with hypercoagulability [54]. Interestingly, some of these factors’ associations were also found in CD. Therefore, the increased risk of thromboembolism in IBD (which has been shown in multiple studies), the increased prevalence of IBD in CD, and the autoimmune nature of both disorders support the proposed statement of an increased risk of thromboembolism in CD.

Patients with CD have higher levels of antiphospholipid antibodies compared to non-CD patients [55]. Our results show that antiphospholipid antibodies were present in 10.5% of patients. Interestingly, studies show that the level of antiphospholipid antibodies did not decrease after the initiation of a GFD. This observation suggests that the formation of antibodies is not triggered by gluten, but might be related to the autoimmune nature of CD itself [56].

In the pathogenesis of thrombosis, changes in platelet counts could be considered as one of the most important factors. According to our results, thrombocytosis was more commonly observed than thrombocytopenia (Table 1). Although TEs in secondary thrombocytosis are rare [57], there are some cases describing patients with TE secondary to it [58,59]. The etiology of thrombocytosis is complex, and includes several different factors, such as chronic inflammation, hyposplenism and iron deficiency anemia [5,6]. It has been clearly established that elevated endogenous levels of cytokines in patients with chronic inflammatory states lead to thrombocytosis [5,6]. Additionally, hyposplenism, which is presumably caused by antibody deposits in the spleen, could be another cause of thrombocytosis [5,6]. Lastly, elevated serum erythropoietin in IDA leads to the proliferation of common progenitor cells in the bone marrow, resulting in an elevated platelet count (57). One of the mechanisms of thrombus generation in IDA is transferrin up-regulation, since transferrin potentiates thrombin and FXIIa, and blocks the inactivation of coagulation proteases by antithrombin [60,61].

On the other hand, thrombocytopenia is also observed in CD, and is usually immune-mediated [62]. Even though the platelet count is decreased in such cases, immune-mediated thrombocytopenia can be considered as a pro-thrombotic state, as microparticles shed from platelets have prothrombotic features, due to phosphatidylserine exposure and high levels of tissue factor expression [63].

Dehydration and hyperviscosity, caused by profuse diarrhea, could be considered as significant risk factors for thrombosis in this group of patients, particularly in regard to cerebral venous sinus thrombosis [64]; however, the presence of TEs in patients who lack any GI symptoms argues against this hypothesis as the sole reason for VTE development.

Finally, there are several conditions that are not specifically associated with CD, but, if present, could predispose that individual to a TE, for example, some cases of inherited thrombophilia (PT20210, FV Leiden), malignancies (myeloproliferative neoplasms—JAK2 mutation), pregnancy, immobilization, or the use of contraceptive pills. All of these conditions may have additive effects with the aforementioned factors, which are known to be the consequence of CD. For that reason, risk stratification for thrombosis development and thrombophilia screening should be considered in high-risk CD patients.

### 4.5. Prevention, Treatment, and Outcome

Strict life-long adherence to a GFD is the mainstay for the treatment of CD, and the prevention of complications, including VTE. This is evidenced by a significantly decreased risk of VTE after a 1-year follow-up [64,65,66]. Some of the patients included in our review were not adherent to a GFD at the time of thrombosis occurrence [34,67,68]. Conversely, in other patients, the initiation of a GFD led to protein C, protein S and homocysteine level normalization [40,69,70]. This could be because of the reduction in intestinal inflammation, and because the recovery of the mucosal layer leads to appropriate reabsorption of nutrients (B12, B9, and B6), normalization of homocysteine and iron levels, and reduced production of pro-thrombogenic cytokines, all of which significantly decrease the risk of thrombosis [71]. Most of the patients in our review favorably responded to GFD implementation/reinstitution, and experienced resolution of various CD manifestations, including thrombosis [9,71,72]. However, in certain cases, clinical benefit was not evident, and two patients experienced cerebral thrombosis, despite commencing and being compliant with a GFD [73]. However, those patients were treated with vitamin K.

The role of anticoagulation in CD, especially in terms of prophylaxis, has not been investigated, and further studies are needed to assess the risk and benefits of this therapy [66]. In our review, 69% of the patients received anticoagulation therapy, and vitamin K antagonists were most commonly instituted. Our review has found anticoagulation therapy to be remarkably important, with a 100% survival rate of the patients who received anticoagulants. This indicates that anticoagulation treatment should be started in all CD patients with thrombosis, unless there are strict contraindications. However, the required duration of therapy remains unclear and should be answered in more rigorous prospective studies in the future.

Patients who adhere to a GFD usually have a good outcome, but their overall mortality risk is still increased [74,75]. Our study demonstrated a mortality rate of 14.5%, which is slightly higher than the previously reported mortality rate of 13.2% [74]. The risk is the highest in the first year after CD diagnosis and stays elevated during a 10-year follow-up [74]. Death usually occurs due to complications, with the most common complications being cardiovascular disease, malignancy (e.g., non-Hodgkin lymphoma), respiratory problems (e.g., pneumonia), and liver disease [74,76].

### 4.6. Limitations of the Study

The limitations of this study are related to the inherent nature of systematic reviews. We additionally recognize that all cases included were written in the English language, so we might have missed some high-quality case reports published in languages other than English. Our cases were selected from the journals indexed only in the PubMed database. While we focused only on PubMed-indexed peer-reviewed journals to avoid low-quality case reports, we acknowledge that this approach might inadvertently miss some high-quality reports. Finally, due to the lack of access for the authors who performed the search, other database searches were not possible, and this is another notable limitation. Lastly, one should bear in mind the existence of publication bias, since peculiar cases are more likely to be published.

## 5. Conclusions

This review summarized 55 cases of TEs in patients with CD. TEs in CD seem to be more frequent in females, and while in 41.3% of cases there is another potential cause of thrombosis, CD as a sole risk factor was present in 58.7%. While TEs can be diagnosed following CD diagnosis, they more frequently proceed the diagnosis or are diagnosed concurrently. Hence, we recommend universal testing for CD in all patients with new diagnosis of VTE, provoked or unprovoked. VTE in CD should be treated with anticoagulation in the absence of contraindication (however, the duration of this treatment remains unclear), in conjunction with the utility of antiplatelet agents and a GFD in primary prophylaxis. Future prospective studies are needed to determine the exact causality between CD and VTE, the cost effectiveness of screening for CD in patients during their first episode of VTE, and treatment modality and duration.

## Figures and Tables

**Table 1:** Demographics, clinical presentation and risk factors for thrombosis in reported cases of thrombotic events in celiac disease.

Demographic Characteristics			
**Gender**		**Median age (years)**	**Age range (years)**
Female	34 (61.82%)	29.5	8–59
Male	21 (38.18%)	34	15–66
Total	55 (100%)	32	8–66
**Geographical distribution**			
Africa	18 (32.73%)		
Asia	15 (27.27%)		
Australia	2 (3.64%)		
Europe	17 (30.91%)		
North America	3 (5.45%)		
**Order of diagnosis**			
CD first	17 (30.91%)		
Thrombosis first	19 (34.54%)		
At the same time	19 (34.54%)		
**Presenting signs and symptoms**			
Abdominal symptoms (distension, pain, diarrhea, vomiting, etc.)	26/55 (47.27%)		
Neurological symptoms and/or altered vision	12/55 (21.81%)		
Generalized weakness and fatigue	9/55 (16.36%)		
Lower extremities swelling	8/55 (14.54%)		
Weight loss/growth restriction	6/55 (10.91%)		
Anemia	4/55 (7.27%)		
Bleeding (hematochezia, hemoptysis)	2/55 (3.63%)		
Other	7/55 (12.72%)		
Not reported	6/55 (10.91%)		
**Comorbidities**			
Yes	19 (34.54%)		
No	36 (65.45%)		
**Laboratory parameters**			
**Anemia**			
Yes	29 (52.72%)		
No	8 (14.54%)		
Not reported	18 (32.72%)		
**Platelet count**			
Increased	6 (10.91%)		
Decreased	4 (7.27%)		
Within normal range	19 (34.54%)		
Not reported	26 (47.27%)		
**Thrombosis risk factors**			
Inflammation	7/55 (12.72%)		
Oral contraceptives	5/55 (9.09%)		
Immobilization	3/55 (5.45%)		
Dehydration	3/55 (5.45%)		
Malignancy	3/55 (5.45%)		
Pregnancy	2/55 (3.63%)		
Surgery	2/55 (3.63%)		
No risk factors	12/55 (21.81%)		
Not reported	24/55 (43.63%)		
**Thrombophilia**			
**Yes**	19 (34.54%)		
Hyperhomocysteinemia	7/19 (36.84%)		
Protein S deficiency	6/19 (31.57%)		
Protein C deficiency	6/19 (31.57%)		
Antiphospholipid antibodies	2/19 (10.52%)		
APCR	1/19 (5.26%)		
Factor V Leiden	2/19 (10.52%)		
PAI–1 4G/5G polymorphism	1/19 (5.26%)		
PT20210 mutation	1/19 (5.26%)		
**No**	27 (49.09%)		
**Not specified**	9 (16.36%)		

CD—celiac disease; APCR—activated protein C resistance; PAI—plasminogen activator inhibitor; PT—prothrombin.

**Table 2:** Thrombosis characteristics, treatment and patients’ outcome.

Type of Thrombosis	
Venous	44 (80%)
Arterial	9 (16.36%)
Both	2 (3.64%)
**Number of sites involved**	
1	43 (78.18%)
2	10 (18.18%)
3 or more	2 (3.64%)
**Site of thrombosis**	
Hepatic veins	17/55 (30.91%)
Deep venous thrombosis of lower extremities	10/55 (18.18%)
Cerebral blood vessels	10/55 (18.18%)
Pulmonary thromboembolism	9/55 (16.36%)
Portal system vessels	8/55 (14.54%)
Central retinal vein	4/55 (7.27%)
Abdominal arteries	3/55 (5.45%)
Other	5/55 (9.09%)
**Therapy**	
Yes	38 (69.09%)
Vitamin K antagonists	19/38 (50%)
Heparin/LMWH	15/38 (39.47%)
Antiplatelet medications	5/38 (13.15%)
Direct-acting anticoagulants	1/38 (2.63%)
Oral anticoagulants (not specified)	8/38 (21.05%)
No	17 (30.91%)
**Outcome**	
Recovered	29 (52.72%)
Partially recovered	14 (25.45%)
Death	8 (14.54%)
Thrombosis extension	2 (3.64%)
Unknown	2 (3.64%)

LMWH—low-molecular-weight heparin.

## Data Availability

All data are publicly available on the PubMed database.
